# Supplementary material for: Causal Relationship between Aging and Anorexia Nervosa: A White-Matter-Microstructure-Mediated Mendelian Randomization Analysis
Source: Biomedicines. 2024 Aug 16;12(8):1874. doi: 10.3390/biomedicines12081874 (PMC11351342; doi:10.3390/biomedicines12081874)
Supplement: Supplementary file 1 [file biomedicines-12-01874-s001.zip › Supplementary Figure/Figure S4/Singe SNP plot of MR analysis for TL to ubm-b-2118.pdf]

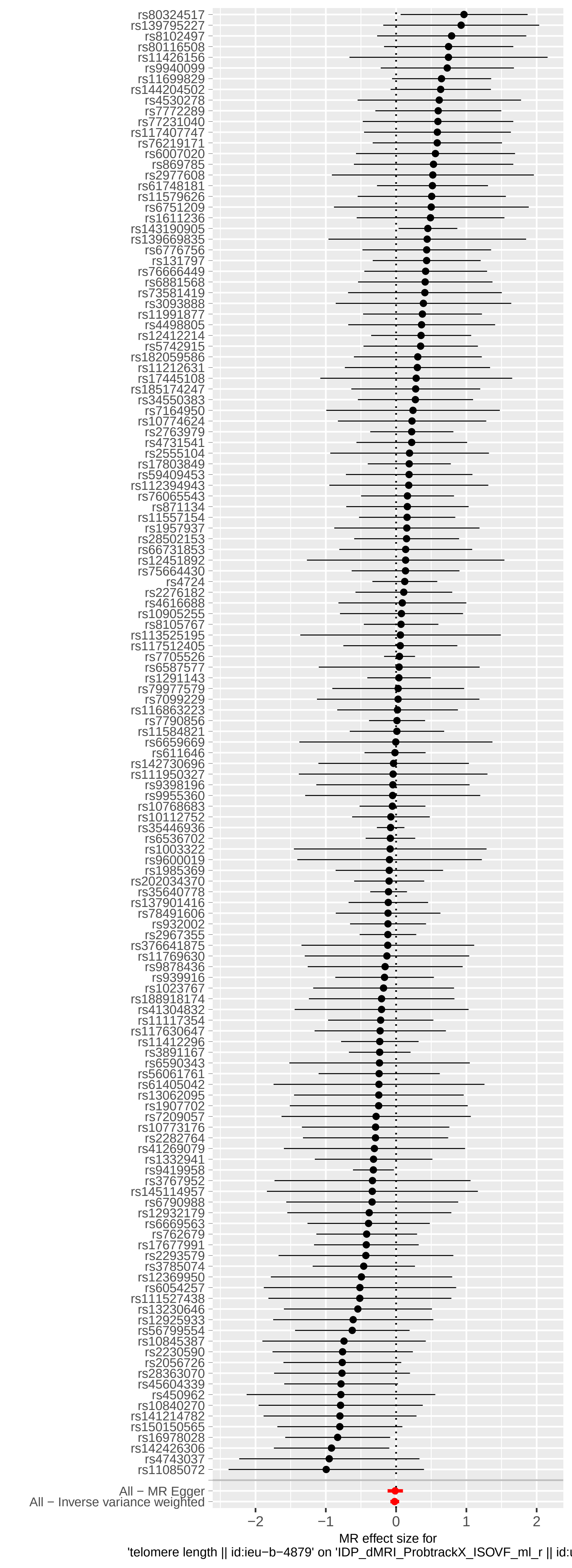
rs80324517
rs139795227
rs8102497
rs80116508
rs11426156
rs9940099
rs11699829
rs144204502
rs4530278
rs7772289
rs77231040
rs117407747
rs76219171
rs6007020
rs869785
rs2977608
rs61748181
rs11579626
rs6751209
rs1611236
rs143190905
rs139669835
rs6776756
rs131797
rs76666449
rs6881568
rs73581419
rs3093888
rs11991877
rs4498805
rs12412214
rs5742915
rs182059586
rs11212631
rs17445108
rs185174247
rs34550383
rs7164950
rs10774624
rs2763979
rs4731541
rs2555104
rs17803849
rs59409453
rs112394943
rs76065543
rs871134
rs11557154
rs1957937
rs28502153
rs66731853
rs12451892
rs75664430
rs4724
rs2276182
rs4616688
rs10905255
rs8105767
rs113525195
rs117512405
rs7705526
rs6587577
rs1291143
rs79977579
rs7099229
rs116863223
rs7790856
rs11584821
rs6659669
rs611646
rs142730696
rs111950327
rs9398196
rs9955360
rs10768683
rs10112752
rs35446936
rs6536702
rs1003322
rs9600019
rs1985369
rs202034370
rs35640778
rs137901416
rs78491606
rs932002
rs2967355
rs376641875
rs11769630
rs9878436
rs939916
rs1023767
rs188918174
rs41304832
rs11117354
rs117630647
rs11412296
rs3891167
rs6590343
rs56061761
rs61405042
rs13062095
rs1907702
rs7209057
rs10773176
rs2282764
rs41269079
rs1332941
rs9419958
rs3767952
rs145114957
rs6790988
rs12932179
rs6669563
rs762679
rs17677991
rs2293579
rs3785074
rs12369950
rs6054257
rs111527438
rs13230646
rs12925933
rs56799554
rs10845387
rs2230590
rs2056726
rs28363070
rs45604339
rs450962
rs10840270
rs141214782
rs150150565
rs16978028
rs142426306
rs4743037
rs11085072
All − MR Egger
All − Inverse variance weighted
−2
−1
0
1
2
MR effect size for
'telomere length || id:ieu−b−4879' on 'IDP_dMRI_ProbtrackX_ISOVF_ml_r || id:u
